# Crystal structure of (2-amino-7-methyl-4-oxido­pteridine-6-carboxyl­ato-κ^3^ *O* ^4^,*N* ^5^,*O* ^6^)aqua­(1,10-phenanthroline-κ^2^ *N*,*N*′)copper(II) trihydrate

**DOI:** 10.1107/S1600536814022302

**Published:** 2014-10-18

**Authors:** Siddhartha S. Baisya, Parag S. Roy

**Affiliations:** a Department of Chemistry, University of North Bengal, Siliguri 734 013, India

**Keywords:** pterin, copper, π–π stacking, crystal structure

## Abstract

In the title compound, [Cu(C_8_H_5_N_5_O_3_)(C_12_H_8_N_2_)(H_2_O)]·3H_2_O, the Cu^II^ cation is *O*,*N*,*O*′-chelated by the 2-amino-7-methyl-4-oxidopteridine-6-carboxyl­ate anion and *N*,*N*′-chelated by the 1,10-phenanthroline (phen) ligand. A water mol­ecule further coordinates to the Cu^II^ cation to complete the elongated distorted octa­hedral coordination geometry. In the mol­ecule, the pteridine ring system is essentially planar [maximum deviation = 0.055 (4) Å], and its mean plane is nearly perpendicular to the phen ring system [dihedral angle = 85.97 (3)°]. In the crystal, N—H⋯O, O—H⋯N and O—H⋯·O hydrogen bonds, as well as weak C—H⋯O hydrogen bonds and C—H⋯π inter­actions, link the complex mol­ecules and lattice water mol­ecules into a three-dimensional supra­molecular architecture. Extensive π–π stacking between nearly parallel aromatic rings of adjacent mol­ecules are also observed, the centroid-to-centroid distances being 3.352 (2), 3.546 (3), 3.706 (3) and 3.744 (3) Å.

## Abstract 2

In a hydrated copper(II) complex, 2-amino-7-methyl-4-oxidopteridine-6-carboxyl­ate and 1,10-phenanthroline ligands chelate the Cu^II^ cation while a water mol­ecule further coordinates to the Cu^II^ cation to complete the elongated distorted octa­hedral coordination geometry.

## Chemical context   

The ubiquitous presence of pterins in nature including several classes of metalloenzymes, has catalysed developments of their coordination chemistry (Basu & Burgmayer, 2011 ▶; Burgmayer, 1998 ▶; Dix & Benkovic, 1988 ▶; Erlandsen *et al.*, 2000 ▶; Fitzpatrick, 2003 ▶). Literature survey reveals the paucity of structurally characterized Cu^II^ complexes involving tridentate pterin coordination (Kohzuma *et al.*, 1989 ▶). The present work is concerned with the title complex, possessing both a tridentate pterin ligand and a π-acidic ligand like phen.
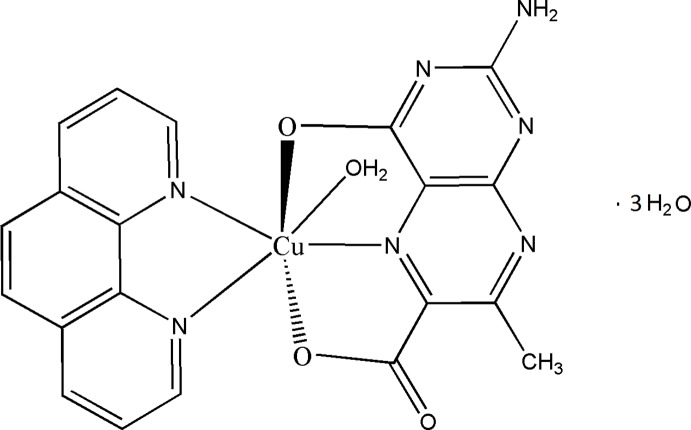


## Structural commentary   

**Figure 1:**
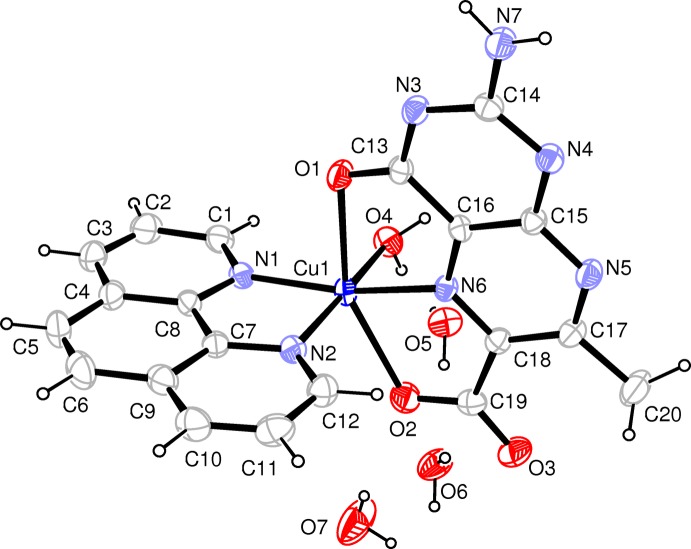
The mol­ecular structure of the title compound. Displacement ellipsoids are drawn at the 30% probability level.

The hexa­coordinated Cu^II^ atom is located in an axially elongated distorted octa­hedron (Fig. 1 ▶ and Table 1 ▶). The equatorial plane is formed by the two N atoms of phen, the pyrazine ring N atom of the pterin ligand and the aqua O atom. The axial positions are occupied by the two pterin O atoms, with the former one forming the longest axial bond [2.384 (3) Å]. Apart from the characteristic Jahn–Teller effect, another reason for distortion from a regular octa­hedral geometry is that the pterin ligand forms two five-membered chelate rings with small bite angles [76.47 (10) and 74.66 (11)°]. Consideration of the charge balance of this complex indicates that this pterin ligand acts as a binegative tridentate *O*,*N*,*O*′-donor. A near orthogonal disposition of the phen ligand and pterin chelate ring helps to minimize the steric repulsion. Of the three axes, the least deviation from linearity is observed in the O4—Cu1—N2 direction [174.45 (13)°]. Location of the pyrazine ring N atom (N6) in the equatorial plane is in agreement with earlier observations on related copper and cobalt complexes (Baisya *et al.*, 2013 ▶; Odani *et al.*, 1992 ▶); the Cu1—N6 bond length [1.999 (3) Å] is the shortest one in this case.

The multiple bond character of the O1—C13 bond [1.237 (4) Å] may be elucidated in the light of Joule’s hypothesis (Beddoes *et al.*, 1993 ▶; Russell *et al.*, 1992 ▶), suggesting electron-density withdrawal from the pyrazine ring N5 by the pyrimidine ring C13 carbonyl group through mesomeric inter­action. Formation of the O1—Cu1 bond assists this electron flow towards atom O1, with possible participation of the electron-rich N7—C14 [1.327 (5) Å] bond in this process.

## Supra­molecular features   

**Figure 2:**
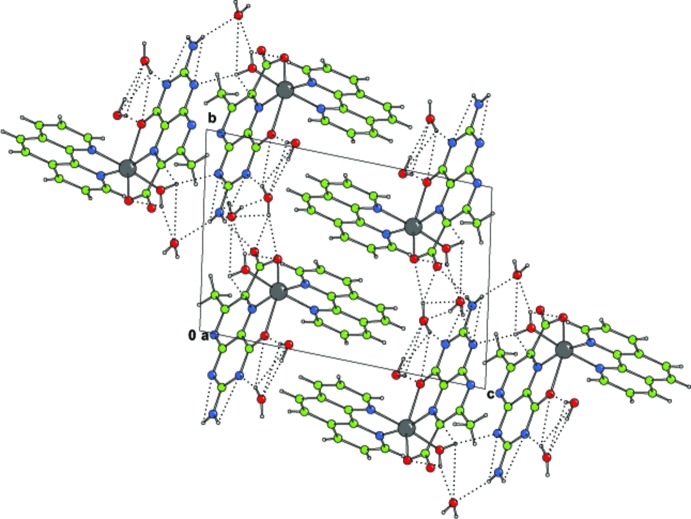
The crystal packing diagram of the title compound, viewed along the *a* axis. Hydrogen bonds (dotted lines) assist the formation of a layer structure parallel to (001).

**Figure 3:**
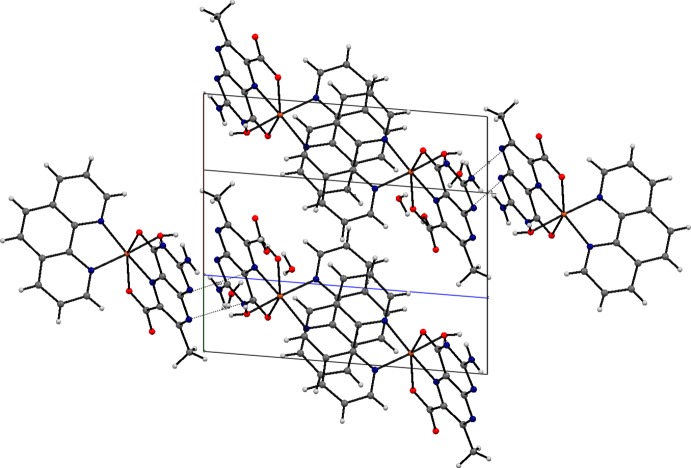
A mol­ecular packing diagram highlighting π–π stacking inter­actions between neighbouring phen–phen and pterin–pterin rings.

In the crystal, inter­molecular N—H⋯·O, O—H⋯·N and O—H⋯O hydrogen bonds (Table 2 ▶) link the complex mol­ecules and lattice water mol­ecules into a layer parallel to (001) (Fig. 2 ▶). Inter­molecular weak C—H⋯O hydrogen bonds and C—H⋯π inter­actions are also observed in the crystal. In addition, π–π stacking between nearly parallel pterin ring systems of adjacent mol­ecules occurs in the crystal structure, the centroid–centroid distance being 3.352 (2) Å (Fig. 3 ▶). Again, the nearly parallel phen rings of adjacent mol­ecules also display π–π stacking inter­actions with centroids distances of 3.546 (3), 3.706 (3) and 3.744 (3) Å. These inter­molecular inter­actions link the mol­ecules into a three-dimensional supra­molecular architecture.

## Database survey   

The crystal structures of copper(II) complexes chelated by the pterin-6-carboxyl­ate anion have been reported by Kohzuma *et al.* (1989 ▶) and Funahashi *et al.* (1999 ▶). In both complexes, the Cu^II^ atom has the elongated distorted octa­hedral coordination geometry.

## Synthesis and crystallization   

2-Amino-4-hy­droxy-7-methyl­pteridine-6-carb­oxy­lic acid sesquihydrate (C_8_H_7_N_5_O_3_·1.5H_2_O) was obtained by a published procedure (Wittle *et al.*, 1947 ▶). The title complex could be obtained by two different methods; the crystals obtained by method B have been used for the present structural study. The X-ray structural data of the crystals synthesized by method A, are available from the Cambridge Crystallographic Data Center (CCDC deposition No. 985054).

**Method A**. The title complex was synthesized by bubbling ­oxy­gen into an aqueous reaction mixture (50 ml) containing Cu(NO_3_)_2_·3H_2_O (30 mg, 0.125 mmol), 1,10-phenanthroline monohydrate (25 mg, 0.125 mmol) and pterin (31 mg, 0.125 mmol) dissolved in NaOH (11 mg, 0.275 mmol) for 60 h at 310–312 K under subdued light; additional NaOH solution was added for adjusting the initial pH at 10.5. Within a short while the initial bright-green solution turned hazy blue due to the presence of a fine white precipitate which gradually disappeared substanti­ally. The final blue solution was slightly hazy. Upon storage under aerobic conditions for one week the clear blue solution yielded green crystals, suitable for X-ray structure determination. Analysis calculated for C_20_H_21_CuN_7_O_7_: C 44.90, H 3.93, N 18.33%; found: C 44.38, H 4.06, N 17.65%. ESIMS data: the mol­ecular ion peak [*M* + 2H]^+^ appeared at 536.4 (relative abundance = 41.2%); the [*M* − 4H_2_O − 3H]^+^ peak was observed at 459.2 (relative abundance = 100%), indicating stability of the desolvated ternary species arising from the title complex.

**Method B**. Using NaBH_4_ reduction in equimolar proportion of the original complex (obtained by **Method A**) and subsequent aerial reoxidation of the reduced complex to the present crystals merits attention due to the involvement of intricate redox chemistry. The NaBH_4_ treatment (Beddoes *et al.*, 1993 ▶; Russell *et al.*, 1992 ▶) leads to the formation of a dark-brown compound in solution, which could be isolated in the solid state and characterized (microanalysis, ESIMS, 2DNMR, *etc.*,) to be Na_2_[Cu_2_^I^(*L*′)_2_(phen)(H_2_O)_4_]·2H_2_O, where *L*′ is the 7,8-di­hydro form of the present pterin ligand anion (C_8_H_5_N_5_O_3_) (Burgmayer, 1998 ▶); it is able to convert bromo­benzene into 4-bromo­phenol in the presence of ­oxy­gen (Baisya & Roy, unpublished results). However, in the absence of any substrate (*e.g.* bromo­benzene; Dix & Benkovic, 1988 ▶), aerial oxidation reconverts the reduced compound to the title complex (**Method B**).

Although the title compound could be obtained by two alternative methods, the present structural data obtained using the crystals from **Method B**, represent better accuracy [*R* = 0.057 and *wR*(*F* ^2^)= 0.135] as compared to the other one [*R* = 0.113 and *wR*(*F* ^2^) = 0.279].

Cyclic voltammetry data of this complex indicate an *E*°′ value of −0.68 V; now using an *E*°′ value of −0.80 V for NaBH_4_ in neutral medium (Chatenet *et al.*, 2006 ▶; Celikkan *et al.*, 2007 ▶), an *E*_cell_ value (*E*_cell_ = *E*_1_ − *E*_2_; Segel, 1976 ▶) of 0.12 V is obtained for the Cu^II^ → Cu^I^ reduction in the title complex; it is within the range of *E*_cell_ value (0.033 V) for the Fe^III^–tetra­hydro­biopterin reduction in phenyl­alanine hy­droxy­lase (Hagedoorn *et al.*, 2001 ▶; Gorren *et al.*, 2001 ▶). The dark-brown reduced complex (as above) shows an *E*°′ value of −0.67 V (cyclic voltammetry); using an *E*°′ value of 0.70 V for the O_2_/H_2_O_2_ couple, an *E*_cell_ value of 1.37 V is obtained, indicating facile aerial oxidation. Now using an *E*°′ value of 0.19 V for the chelated pterin ligand (oxidized/aromatic; Eberlein *et al.*, 1984 ▶), synchronization of its reduction or oxidation with the above redox process may be rationalized. Actually, for pterin-containing metalloenzymes the redox processes at the metal centres could be linked to the changes in the pterin ring oxidation level (Burgmayer, 1998 ▶; Erlandsen *et al.*, 2000 ▶).

## Refinement   

Crystal data, data collection and structure refinement details are summarized in Table 3 ▶. H atoms attached to N and O atoms were located in a difference Fourier map and refined with distance constraints of N—H = 0.86 (1) Å and O—H = 0.82 (1) Å. H atoms attached to C atoms were positioned geometrically, with C—H = 0.93–0.96 Å, and refined in riding mode. For all atoms, *U*_iso_(H) = 1.2–1.5*U*_eq_(C,N,O).

## Supplementary Material

Crystal structure: contains datablock(s) I, New_Global_Publ_Block. DOI: 10.1107/S1600536814022302/xu5822sup1.cif

Structure factors: contains datablock(s) I. DOI: 10.1107/S1600536814022302/xu5822Isup2.hkl

CCDC reference: 1028413

Additional supporting information:  crystallographic information; 3D view; checkCIF report

## Figures and Tables

**Table 1:** Selected bond lengths ()

Cu1N1	2.002(3)	Cu1O1	2.384(3)
Cu1N2	2.037(3)	Cu1O2	2.304(3)
Cu1N6	1.999(3)	Cu1O4	2.019(3)

**Table 2:** Hydrogen-bond geometry (, ) *Cg* is the centroid of the N3/N4/C13C16 ring.

*D*H*A*	*D*H	H*A*	*D* *A*	*D*H*A*
O4H4*C*O5	0.82(3)	1.92(3)	2.722(4)	169(5)
O4H4*D*N4^i^	0.81(3)	2.26(3)	3.038(4)	161(5)
O5H5*C*O6	0.82(3)	1.96(4)	2.748(5)	162(4)
O5H5*D*N4^ii^	0.82(5)	2.07(5)	2.891(5)	176(3)
O6H6*C*O2	0.82(3)	2.23(3)	2.921(4)	141(5)
O6H6*C*O3	0.82(3)	2.25(4)	3.029(4)	158(5)
O7H7*C*O6	0.82(2)	2.24(3)	2.965(6)	148(5)
O7H7*D*O1^iii^	0.81(5)	2.16(4)	2.943(6)	162(5)
N7H7*E*O5^i^	0.85(5)	2.17(4)	2.998(6)	162(4)
N7H7*F*O3^iv^	0.86(4)	2.14(5)	2.908(5)	148(4)
C1H1O3^v^	0.93	2.47	3.175(6)	133
C10H10O1^vi^	0.93	2.54	3.406(5)	155
C12H12O7^vii^	0.93	2.57	3.343(7)	140
C6H6*Cg* ^vi^	0.93	2.82	3.740(5)	173

Symmetry codes: (i) 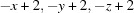; (ii) 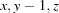; (iii) 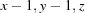; (iv) 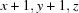; (v) 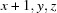; (vi) 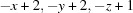; (vii) 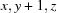.

**Table 3:** Experimental details

Crystal data	
Chemical formula	[Cu(C_8_H_5_N_5_O_3_)(C_12_H_8_N_2_)(H_2_O)]3H_2_O
*M*_r_	534.98
Crystal system, space group	Triclinic, *P* 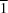
Temperature (K)	273
*a*, *b*, *c* ()	8.5399(17), 10.038(2), 13.601(3)
, , ()	97.292(3), 94.587(3), 110.999(3)
*V* (^3^)	1069.8(4)
*Z*	2
Radiation type	Mo *K*
(mm^1^)	1.08
Crystal size (mm)	0.20 0.05 0.03

Data collection	
Diffractometer	Bruker Kappa APEXII
Absorption correction	Multi-scan (*SADABS*; Bruker, 2001 ▶)
*T*_min_, *T*_max_	0.813, 0.968
No. of measured, independent and observed [*I* > 2(*I*)] reflections	8227, 4134, 3590
*R*_int_	0.024
(sin /)_max_ (^1^)	0.617

Refinement	
*R*[*F* ^2^ > 2(*F* ^2^)], *wR*(*F* ^2^), *S*	0.051, 0.136, 1.15
No. of reflections	4134
No. of parameters	349
No. of restraints	10
H-atom treatment	H atoms treated by a mixture of independent and constrained refinement
_max_, _min_ (e ^3^)	0.66, 0.31

Computer programs: *APEX2* and *SAINT* (Bruker, 2007 ▶), *SHELXS97* (Sheldrick, 2008 ▶), *CRYSTALS* (Betteridge *et al.*, 2003 ▶) and *CAMERON* (Watkin *et al.*, 1996 ▶).

## supplementary crystallographic information

### Crystal data

**Table:**

[Cu(C_8_H_5_N_5_O_3_)(C_12_H_8_N_2_)(H_2_O)]·3H_2_O	*Z* = 2
*M**_r_* = 534.98	*F*(000) = 550
Triclinic, *P*1	*D*_x_ = 1.661 Mg m^−^^3^
Hall symbol: -P 1	Mo *K*α radiation, λ = 0.71073 Å
*a* = 8.5399 (17) Å	Cell parameters from 4804 reflections
*b* = 10.038 (2) Å	θ = 3.0–29.0°
*c* = 13.601 (3) Å	µ = 1.08 mm^−^^1^
α = 97.292 (3)°	*T* = 273 K
β = 94.587 (3)°	Needle, green
γ = 110.999 (3)°	0.20 × 0.05 × 0.03 mm
*V* = 1069.8 (4) Å^3^	

### Data collection

**Table:**

Bruker Kappa APEXII diffractometer	4134 independent reflections
Radiation source: fine-focus sealed tube	3590 reflections with *I* > 2σ(*I*)
Graphite monochromator	*R*_int_ = 0.024
φ & ω scans	θ_max_ = 26.0°, θ_min_ = 1.5°
Absorption correction: multi-scan (*SADABS*; Bruker, 2001)	*h* = −10→10
*T*_min_ = 0.813, *T*_max_ = 0.968	*k* = −12→12
8227 measured reflections	*l* = −16→16

### Refinement

**Table:**

Refinement on *F*^2^	Primary atom site location: structure-invariant direct methods
Least-squares matrix: full	Secondary atom site location: difference Fourier map
*R*[*F*^2^ > 2σ(*F*^2^)] = 0.051	Hydrogen site location: inferred from neighbouring sites
*wR*(*F*^2^) = 0.136	H atoms treated by a mixture of independent and constrained refinement
*S* = 1.15	*w* = 1/[σ^2^(*F*_o_^2^) + (0.052*P*)^2^ + 1.8801*P*] where *P* = (*F*_o_^2^ + 2*F*_c_^2^)/3
4134 reflections	(Δ/σ)_max_ = 0.001
349 parameters	Δρ_max_ = 0.66 e Å^−^^3^
10 restraints	Δρ_min_ = −0.31 e Å^−^^3^

### Special details

**Table:**

Experimental. The crystal was placed in the cold stream of an Oxford Cryosystems open-flow nitrogen cryostat (Cosier & Glazer, 1986) with a nominal stability of 0.1 K.Cosier, J. & Glazer, A. M., 1986. J. Appl. Cryst. 105–107.
Geometry. All esds (except the esd in the dihedral angle between two l.s. planes) are estimated using the full covariance matrix. The cell esds are taken into account individually in the estimation of esds in distances, angles and torsion angles; correlations between esds in cell parameters are only used when they are defined by crystal symmetry. An approximate (isotropic) treatment of cell esds is used for estimating esds involving l.s. planes.
Refinement. Refinement of F^2^ against ALL reflections. The weighted R-factor wR and goodness of fit S are based on F^2^, conventional R-factors R are based on F, with F set to zero for negative F^2^. The threshold expression of F^2^ > 2sigma(F^2^) is used only for calculating R-factors(gt) etc. and is not relevant to the choice of reflections for refinement. R-factors based on F^2^ are statistically about twice as large as those based on F, and R- factors based on ALL data will be even larger.

### Fractional atomic coordinates and isotropic or equivalent isotropic displacement parameters (Å^2^)

**Table:**

	*x*	*y*	*z*	*U*_iso_*/*U*_eq_	
Cu1	0.96752 (6)	0.72720 (5)	0.73038 (3)	0.02733 (16)	
O1	1.1883 (3)	0.9602 (3)	0.7732 (2)	0.0353 (7)	
O2	0.6970 (4)	0.5696 (3)	0.7379 (2)	0.0402 (7)	
O3	0.4778 (4)	0.5558 (3)	0.8185 (3)	0.0463 (8)	
O4	1.0500 (4)	0.6534 (3)	0.8475 (2)	0.0356 (7)	
O5	0.8413 (4)	0.4056 (3)	0.9019 (3)	0.0425 (7)	
O6	0.5380 (4)	0.2754 (3)	0.7786 (3)	0.0496 (8)	
O7	0.5000 (6)	−0.0210 (5)	0.6878 (5)	0.0974 (17)	
N1	1.1082 (4)	0.6636 (3)	0.6383 (2)	0.0288 (7)	
N2	0.8823 (4)	0.7835 (4)	0.6039 (2)	0.0296 (7)	
N3	1.2193 (4)	1.1811 (3)	0.8566 (2)	0.0309 (7)	
N4	0.9983 (4)	1.2067 (3)	0.9516 (2)	0.0303 (7)	
N5	0.7502 (4)	1.0056 (4)	0.9482 (3)	0.0330 (8)	
N6	0.8684 (4)	0.8399 (3)	0.8203 (2)	0.0247 (7)	
N7	1.2417 (5)	1.3974 (4)	0.9441 (3)	0.0410 (9)	
C1	1.2221 (5)	0.6068 (5)	0.6587 (3)	0.0360 (9)	
H1	1.2374	0.5846	0.7223	0.043*	
C2	1.3205 (6)	0.5790 (5)	0.5872 (4)	0.0454 (11)	
H2	1.4011	0.5403	0.6039	0.054*	
C3	1.2988 (6)	0.6084 (5)	0.4932 (4)	0.0450 (11)	
H3	1.3617	0.5871	0.4452	0.054*	
C4	1.1804 (5)	0.6712 (5)	0.4691 (3)	0.0374 (10)	
C5	1.1463 (6)	0.7074 (6)	0.3733 (3)	0.0502 (12)	
H5	1.2072	0.6912	0.3224	0.060*	
C6	1.0283 (7)	0.7643 (5)	0.3551 (3)	0.0485 (12)	
H6	1.0084	0.7855	0.2918	0.058*	
C7	0.9651 (5)	0.7606 (4)	0.5263 (3)	0.0291 (8)	
C8	1.0885 (5)	0.6975 (4)	0.5453 (3)	0.0295 (8)	
C9	0.9327 (6)	0.7929 (5)	0.4312 (3)	0.0384 (10)	
C10	0.8072 (6)	0.8508 (5)	0.4183 (3)	0.0449 (11)	
H10	0.7811	0.8741	0.3566	0.054*	
C11	0.7228 (6)	0.8730 (5)	0.4962 (4)	0.0459 (11)	
H11	0.6386	0.9106	0.4877	0.055*	
C12	0.7642 (5)	0.8386 (5)	0.5887 (3)	0.0376 (10)	
H12	0.7070	0.8550	0.6415	0.045*	
C13	1.1328 (5)	1.0386 (4)	0.8256 (3)	0.0275 (8)	
C14	1.1494 (5)	1.2573 (4)	0.9168 (3)	0.0298 (8)	
C15	0.9039 (5)	1.0639 (4)	0.9189 (3)	0.0276 (8)	
C16	0.9640 (5)	0.9774 (4)	0.8548 (3)	0.0245 (8)	
C17	0.6568 (5)	0.8674 (4)	0.9142 (3)	0.0330 (9)	
C18	0.7141 (5)	0.7807 (4)	0.8461 (3)	0.0274 (8)	
C19	0.6205 (5)	0.6222 (4)	0.7984 (3)	0.0316 (9)	
C20	0.4884 (6)	0.8090 (5)	0.9510 (4)	0.0537 (13)	
H20A	0.4874	0.8722	1.0100	0.081*	
H20B	0.4695	0.7143	0.9666	0.081*	
H20C	0.4006	0.8032	0.9000	0.081*	
H4C	0.984 (5)	0.575 (3)	0.856 (4)	0.050*	
H4D	1.062 (6)	0.702 (5)	0.9021 (19)	0.050*	
H5C	0.750 (3)	0.351 (4)	0.871 (3)	0.046 (15)*	
H5D	0.882 (6)	0.346 (4)	0.915 (4)	0.054 (16)*	
H6C	0.540 (6)	0.358 (2)	0.778 (4)	0.050*	
H6D	0.448 (3)	0.215 (4)	0.750 (3)	0.050*	
H7C	0.548 (6)	0.0668 (15)	0.705 (4)	0.050*	
H7D	0.415 (4)	−0.043 (6)	0.715 (4)	0.050*	
H7E	1.204 (6)	1.455 (4)	0.977 (3)	0.050*	
H7F	1.338 (3)	1.442 (5)	0.926 (4)	0.050*	

### Atomic displacement parameters (Å^2^)

**Table:**

	*U*^11^	*U*^22^	*U*^33^	*U*^12^	*U*^13^	*U*^23^
Cu1	0.0323 (3)	0.0302 (3)	0.0238 (3)	0.0159 (2)	0.00750 (18)	0.00499 (18)
O1	0.0324 (15)	0.0302 (15)	0.0431 (17)	0.0104 (12)	0.0163 (13)	0.0017 (12)
O2	0.0420 (17)	0.0321 (16)	0.0416 (17)	0.0097 (13)	0.0077 (14)	0.0001 (13)
O3	0.0329 (16)	0.0410 (18)	0.055 (2)	0.0011 (14)	0.0115 (14)	0.0083 (15)
O4	0.0413 (17)	0.0350 (17)	0.0323 (16)	0.0151 (14)	0.0047 (13)	0.0092 (13)
O5	0.0405 (19)	0.0339 (18)	0.053 (2)	0.0139 (15)	0.0021 (16)	0.0098 (15)
O6	0.0364 (17)	0.0333 (17)	0.076 (3)	0.0082 (14)	0.0124 (17)	0.0077 (17)
O7	0.073 (3)	0.065 (3)	0.155 (5)	0.025 (3)	0.057 (3)	−0.004 (3)
N1	0.0300 (17)	0.0279 (17)	0.0277 (17)	0.0113 (14)	0.0037 (13)	0.0000 (13)
N2	0.0300 (17)	0.0322 (18)	0.0267 (17)	0.0114 (14)	0.0043 (13)	0.0058 (13)
N3	0.0299 (17)	0.0273 (17)	0.0348 (18)	0.0083 (14)	0.0109 (14)	0.0058 (14)
N4	0.0328 (18)	0.0244 (16)	0.0339 (18)	0.0108 (14)	0.0080 (14)	0.0029 (13)
N5	0.0303 (18)	0.0308 (18)	0.040 (2)	0.0128 (15)	0.0123 (15)	0.0034 (15)
N6	0.0257 (16)	0.0248 (16)	0.0249 (16)	0.0090 (13)	0.0077 (13)	0.0069 (12)
N7	0.039 (2)	0.0264 (19)	0.050 (2)	0.0044 (16)	0.0169 (18)	−0.0010 (16)
C1	0.035 (2)	0.036 (2)	0.037 (2)	0.0165 (19)	0.0012 (18)	−0.0006 (18)
C2	0.038 (2)	0.046 (3)	0.053 (3)	0.022 (2)	0.004 (2)	−0.005 (2)
C3	0.038 (2)	0.046 (3)	0.046 (3)	0.013 (2)	0.013 (2)	−0.006 (2)
C4	0.036 (2)	0.034 (2)	0.037 (2)	0.0077 (18)	0.0114 (18)	−0.0016 (17)
C5	0.054 (3)	0.060 (3)	0.033 (2)	0.016 (3)	0.018 (2)	0.002 (2)
C6	0.062 (3)	0.053 (3)	0.029 (2)	0.016 (2)	0.011 (2)	0.013 (2)
C7	0.029 (2)	0.0248 (19)	0.029 (2)	0.0055 (16)	0.0053 (16)	0.0025 (15)
C8	0.030 (2)	0.027 (2)	0.026 (2)	0.0059 (16)	0.0054 (16)	−0.0001 (15)
C9	0.041 (2)	0.036 (2)	0.032 (2)	0.0060 (19)	0.0026 (18)	0.0075 (18)
C10	0.048 (3)	0.050 (3)	0.035 (2)	0.014 (2)	0.000 (2)	0.018 (2)
C11	0.038 (2)	0.050 (3)	0.052 (3)	0.017 (2)	0.000 (2)	0.017 (2)
C12	0.038 (2)	0.040 (2)	0.039 (2)	0.019 (2)	0.0067 (19)	0.0082 (19)
C13	0.029 (2)	0.030 (2)	0.0250 (19)	0.0112 (16)	0.0066 (15)	0.0077 (15)
C14	0.032 (2)	0.0254 (19)	0.031 (2)	0.0090 (16)	0.0032 (16)	0.0052 (16)
C15	0.027 (2)	0.0265 (19)	0.029 (2)	0.0104 (16)	0.0052 (15)	0.0047 (15)
C16	0.0288 (19)	0.0242 (19)	0.0216 (18)	0.0105 (16)	0.0056 (15)	0.0044 (14)
C17	0.027 (2)	0.034 (2)	0.039 (2)	0.0114 (17)	0.0088 (17)	0.0074 (17)
C18	0.0260 (19)	0.029 (2)	0.028 (2)	0.0110 (16)	0.0049 (15)	0.0076 (15)
C19	0.031 (2)	0.031 (2)	0.031 (2)	0.0085 (17)	0.0003 (17)	0.0091 (16)
C20	0.036 (3)	0.045 (3)	0.076 (4)	0.009 (2)	0.026 (2)	−0.001 (2)

### Geometric parameters (Å, º)

**Table:**

Cu1—N1	2.002 (3)	N7—H7E	0.856 (10)
Cu1—N2	2.037 (3)	N7—H7F	0.854 (11)
Cu1—N6	1.999 (3)	C1—C2	1.400 (6)
Cu1—O1	2.384 (3)	C1—H1	0.9300
Cu1—O2	2.304 (3)	C2—C3	1.361 (7)
Cu1—O4	2.019 (3)	C2—H2	0.9300
O1—C13	1.237 (5)	C3—C4	1.408 (7)
O2—C19	1.267 (5)	C3—H3	0.9300
O3—C19	1.234 (5)	C4—C8	1.404 (6)
O4—H4C	0.819 (10)	C4—C5	1.432 (7)
O4—H4D	0.812 (10)	C5—C6	1.346 (7)
O5—H5C	0.819 (10)	C5—H5	0.9300
O5—H5D	0.820 (10)	C6—C9	1.430 (7)
O6—H6C	0.823 (10)	C6—H6	0.9300
O6—H6D	0.817 (10)	C7—C9	1.403 (6)
O7—H7C	0.819 (10)	C7—C8	1.433 (6)
O7—H7D	0.815 (10)	C9—C10	1.400 (6)
N1—C1	1.321 (5)	C10—C11	1.367 (7)
N1—C8	1.363 (5)	C10—H10	0.9300
N2—C12	1.328 (5)	C11—C12	1.398 (6)
N2—C7	1.357 (5)	C11—H11	0.9300
N3—C13	1.345 (5)	C12—H12	0.9300
N3—C14	1.364 (5)	C13—C16	1.460 (5)
N4—C14	1.355 (5)	C15—C16	1.405 (5)
N4—C15	1.363 (5)	C17—C18	1.425 (6)
N5—C17	1.326 (5)	C17—C20	1.499 (6)
N5—C15	1.348 (5)	C18—C19	1.528 (5)
N6—C16	1.326 (5)	C20—H20A	0.9600
N6—C18	1.333 (5)	C20—H20B	0.9600
N7—C14	1.327 (5)	C20—H20C	0.9600
			
N6—Cu1—N1	165.66 (13)	C6—C5—H5	119.2
N6—Cu1—O4	91.01 (12)	C4—C5—H5	119.2
N1—Cu1—O4	93.79 (13)	C5—C6—C9	121.4 (4)
N6—Cu1—N2	93.79 (13)	C5—C6—H6	119.3
N1—Cu1—N2	82.20 (13)	C9—C6—H6	119.3
O4—Cu1—N2	174.45 (13)	N2—C7—C9	123.3 (4)
N6—Cu1—O2	74.74 (11)	N2—C7—C8	116.3 (3)
N1—Cu1—O2	118.84 (12)	C9—C7—C8	120.4 (4)
O4—Cu1—O2	88.62 (12)	N1—C8—C4	123.1 (4)
N2—Cu1—O2	89.98 (12)	N1—C8—C7	117.1 (3)
N6—Cu1—O1	76.45 (11)	C4—C8—C7	119.8 (4)
N1—Cu1—O1	89.79 (11)	C10—C9—C7	116.7 (4)
O4—Cu1—O1	93.07 (12)	C10—C9—C6	125.0 (4)
N2—Cu1—O1	90.74 (12)	C7—C9—C6	118.3 (4)
O2—Cu1—O1	151.17 (10)	C11—C10—C9	120.1 (4)
C13—O1—Cu1	107.2 (2)	C11—C10—H10	120.0
C19—O2—Cu1	113.0 (3)	C9—C10—H10	120.0
Cu1—O4—H4C	114 (4)	C10—C11—C12	119.4 (4)
Cu1—O4—H4D	116 (4)	C10—C11—H11	120.3
H4C—O4—H4D	101 (5)	C12—C11—H11	120.3
H5C—O5—H5D	100 (5)	N2—C12—C11	122.4 (4)
H6C—O6—H6D	111 (5)	N2—C12—H12	118.8
H7C—O7—H7D	106 (5)	C11—C12—H12	118.8
C1—N1—C8	118.7 (3)	O1—C13—N3	123.3 (3)
C1—N1—Cu1	128.8 (3)	O1—C13—C16	119.8 (3)
C8—N1—Cu1	112.3 (3)	N3—C13—C16	116.9 (3)
C12—N2—C7	118.2 (3)	N7—C14—N4	116.9 (4)
C12—N2—Cu1	129.9 (3)	N7—C14—N3	115.4 (4)
C7—N2—Cu1	111.9 (3)	N4—C14—N3	127.6 (3)
C13—N3—C14	118.8 (3)	N5—C15—N4	119.1 (3)
C14—N4—C15	115.3 (3)	N5—C15—C16	119.8 (3)
C17—N5—C15	119.0 (3)	N4—C15—C16	121.0 (3)
C16—N6—C18	120.8 (3)	N6—C16—C15	120.5 (3)
C16—N6—Cu1	117.0 (2)	N6—C16—C13	119.4 (3)
C18—N6—Cu1	122.2 (3)	C15—C16—C13	120.1 (3)
C14—N7—H7E	122 (4)	N5—C17—C18	121.4 (3)
C14—N7—H7F	125 (3)	N5—C17—C20	116.2 (4)
H7E—N7—H7F	112 (5)	C18—C17—C20	122.4 (4)
N1—C1—C2	121.7 (4)	N6—C18—C17	118.3 (3)
N1—C1—H1	119.2	N6—C18—C19	114.0 (3)
C2—C1—H1	119.2	C17—C18—C19	127.7 (3)
C3—C2—C1	120.3 (4)	O3—C19—O2	124.7 (4)
C3—C2—H2	119.9	O3—C19—C18	119.5 (4)
C1—C2—H2	119.9	O2—C19—C18	115.8 (3)
C2—C3—C4	119.6 (4)	C17—C20—H20A	109.5
C2—C3—H3	120.2	C17—C20—H20B	109.5
C4—C3—H3	120.2	H20A—C20—H20B	109.5
C8—C4—C3	116.6 (4)	C17—C20—H20C	109.5
C8—C4—C5	118.5 (4)	H20A—C20—H20C	109.5
C3—C4—C5	124.9 (4)	H20B—C20—H20C	109.5
C6—C5—C4	121.6 (4)		

### Hydrogen-bond geometry (Å, º)

Cg is the centroid of the N3/N4/C13–C16 ring.

**Table:**

*D*—H···*A*	*D*—H	H···*A*	*D*···*A*	*D*—H···*A*
O4—H4*C*···O5	0.82 (3)	1.92 (3)	2.722 (4)	169 (5)
O4—H4*D*···N4^i^	0.81 (3)	2.26 (3)	3.038 (4)	161 (5)
O5—H5*C*···O6	0.82 (3)	1.96 (4)	2.748 (5)	162 (4)
O5—H5*D*···N4^ii^	0.82 (5)	2.07 (5)	2.891 (5)	176 (3)
O6—H6*C*···O2	0.82 (3)	2.23 (3)	2.921 (4)	141 (5)
O6—H6*C*···O3	0.82 (3)	2.25 (4)	3.029 (4)	158 (5)
O7—H7*C*···O6	0.82 (2)	2.24 (3)	2.965 (6)	148 (5)
O7—H7*D*···O1^iii^	0.81 (5)	2.16 (4)	2.943 (6)	162 (5)
N7—H7*E*···O5^i^	0.85 (5)	2.17 (4)	2.998 (6)	162 (4)
N7—H7*F*···O3^iv^	0.86 (4)	2.14 (5)	2.908 (5)	148 (4)
C1—H1···O3^v^	0.93	2.47	3.175 (6)	133
C10—H10···O1^vi^	0.93	2.54	3.406 (5)	155
C12—H12···O7^vii^	0.93	2.57	3.343 (7)	140
C6—H6···*Cg*^vi^	0.93	2.82	3.740 (5)	173

Symmetry codes: (i) −*x*+2, −*y*+2, −*z*+2; (ii) *x*, *y*−1, *z*; (iii) *x*−1, *y*−1, *z*; (iv) *x*+1, *y*+1, *z*; (v) *x*+1, *y*, *z*; (vi) −*x*+2, −*y*+2, −*z*+1; (vii) *x*, *y*+1, *z*.
